# Target dIsease-Guided placEbo-contRolled (TIGER) design: a novel method for clinical trials of acupuncture

**DOI:** 10.1186/1745-6215-14-359

**Published:** 2013-10-29

**Authors:** Wenke Zheng, Hui Wang, Li Zhang, Zhaoxiang Bian, Hongcai Shang

**Affiliations:** 1 Research Institute of Traditional Chinese Medicine, Tianjin University of Traditional Chinese Medicine, Tianjin 300193, China; 2 Center for Evidence-Based Medicine, Tianjin University of Traditional Chinese Medicine, Tianjin 300193, China; 3 School of Chinese Medicine, Hong Kong Baptist University, Hong Kong, SAR, China

**Keywords:** Placebo design, Acupuncture, Clinical trials, Target disease

## Abstract

**Background:**

At present, the design of an ideal placebo control in acupuncture studies challenges researchers. Previously devised sham acupuncture techniques have reported various imperfections; therefore, the specific effects of acupuncture cannot be accurately examined in clinical trials primarily because of interferences from the placebo effects.

**Method:**

Guided by evidence-based medicine (EBM) theories, we have made an initial attempt to establish a set of control methods for use in acupuncture studies, which is named the target disease-guided placebo-controlled (TIGER) design. In a trial using the TIGER design, participants suffering simultaneously from a predefined target disease and a pseudo target disease will be recruited and randomized to receive identical acupuncture intervention measures. As a result, the interventions not only appear the same but also produce the same stimulations in both groups. The patients in the treatment group will be informed of the actual target disease that the treatment aims for, whereas patients in the control group will be informed that the treatment is for the pseudotarget disease. It is speculated that no psychological response will be aroused in the control group. During data analysis, changes in the outcome measures of the patients in the control groupreveal the real therapeutic effect of acupuncture, and those of patients in the treatment group show both the real and placebo acupuncture effect. In this article, we explain how to put this design into use in a planned randomized clinical trial of acupuncture for the treatment of migraine.

**Results:**

This approach can eliminate the acupuncture placebo effect in the control group that may confound trial results. It is possible to observe the specific and placebo effects of acupuncture for the target disease separately using the TIGER design.

**Conclusions:**

The proposed TIGER design has limitations. It is designed for clinical studies focusing on the specific effects of acupuncture, and it needs to be tested and verified for practicality and feasibility in various clinical research settings.

## Background

Acupuncture has been used in China and other countries in East Asia for a very long time, and literature has documented its efficacy for many conditions and diseases. Following the introduction of evidence-based medicine (EBM) over the last decade, and amidst the growing awareness that the randomized controlled trial (RCT) is the gold standard for efficacy and safety assessment, there has been heated debate about the scientificity of the previously acclaimed effectiveness of acupuncture therapy. The first clinical trial with acupuncture was published in 1959 [1] and since then a number of studies followed. The trials with acupuncture tried to confirm the scientific value following the strict modern medicine merit. This trend has been further emphasized by the EBM approach. By September 2013, a search on the PubMed database using the text word ‘acupuncture’ in all fields and with the filter ‘clinical trial’ activated yielded a total of 3,326 hits. Whether the Western trial design is good for use in eliciting the real effects of acupuncture remains theoretically controversial [2]. Researchers at home and abroad have carried out a number of exploratory experiments, and concluded that a design that controls the placebo effects involved in acupuncture therapy may serve as a solution. The rationale behind the placebo-controlled design is that the global impact of a medical treatment on the human body is a combination of the real curative effect and the placebo effects mainly caused by psychological factors [3]. Typically, trials with a two-arm placebo-controlled design seek to elicit the same psychological effects in both groups using blinding techniques, and the real effect of treatment can be identified by comparing the final results of the two groups. This design applies well to trials with drugs where placebos with identical appearance and inert ingredients are easy to produce and blinding is practical. However, for a complex medical technique such as acupuncture whose effects largely depend on the acupoints chosen and the patient’s needling sensations (the pursuit for a clear-cut separation of the needling sensation and the curative effects appears to many to be theoretically contradictory [4]), the development of a scientific and rigorous placebo control poses a significant challenge.

It has been proposed that an ideal acupuncture placebo should be indistinguishable from true acupuncture in the appearance and the sensations that it produces, and be devoid of any therapeutic or psychological action [5,6]. In the case of RCTs with acupuncture, the situation is complicated by the fact that both the real and the sham treatment are considered to cause a strong placebo response [7], and some of the sham treatments have been found to induce specific physiological responses [8-10]. Furthermore, many of the currently available acupuncture placebo controls, such as sham acupuncture [11], non-specific site stimulation [12], minimal acupuncture [13], needling with a placebo needle [14], mock transcutaneous electrical nerve stimulation (TENS) or mock electroacupuncture [15,16], and sham laser acupuncture [17], are far different from real acupuncture treatment despite careful control and attempted blinding,thus causing varied needling stimulations and most likely inconsistent placebo effects. In fact, a critique on the currently used sham acupuncture highlighted that none can fulfill the requirements set for a traditional placebo control [18].

Therefore, we propose the target disease-guided placebo-controlled (TIGER) design for use in clinical trials with acupuncture. The key features that distinguish the TIGER design from conventional placebo designs is that real and identical acupuncture treatment will be administered to both groups of participants, thus ensuring both interventions not only appear the same but also produce the same stimulations. Secondly, and most importantly, patients in the control group will be informed that the treatment they receive is targeted for the pseudotarget disease, rather than the actual target disease, and thus no psychological response (placebo effects) such as expectations of the alleviation of the target disease will be aroused. Consequently, it is presumed that changes in the outcome measures of the patients in the control group will reveal the real therapeutic effect of acupuncture. In this paper, we will explain how to put this design into use in the actual clinical trial setting with a case study.

## Methods

### Rationale and design

The proposed TIGER design is based on the hypothesis that the overall therapeutic effect of acupuncture is a combination of the real effect (specific effect) and the placebo effect. The specific acupuncture effect is primarily determined by the acupoints chosen and the needling stimulations provided, and the placebo effect originates from a number of individual, context, and cultural factors [7]. Although unsupported, we believe that patients’ psychological factors contribute the most to the acupuncture placebo effects and therefore use ‘psychological factors’ as an umbrella term.

**Figure 1:**
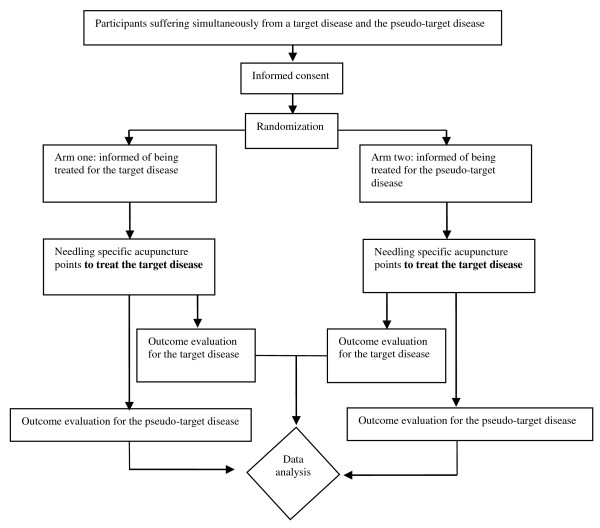
The flow chart of the application of the TIGER design in an acupuncture trial.

In a trial using the TIGER design, participants suffering simultaneously from the target disease and the pseudotarget disease will be recruited, and randomly assigned to two treatment groups (Figure 1). Subsequently, identical intervention measures will be provided in the treatment and control group, thus allowing for the same appearance and sensations that are hard toachieve in currently used placebo methods. However, patients in the treatment group (arm one) will be informed that the treatment is for the target disease, whereas the control group (arm two) will be informed that the treatmentaims for the pseudo target disease, thus eliciting different psychological responses (a key point for this design) in the two groups.

Therefore, distinct from the current practice of blinding all participants of intervention allocation, in the TIGER design one group of patients will be wrongly informed of the target disease that the treatment is for. We believe that this method can curb the confounding psychological responses that will otherwise be aroused in the control group.

We speculate that the results of arm one will show both the real and placebo effect of acupuncture for the target disease, while the results of arm twowill reveal only the real acupuncture effect for the target disease. Similarly, for the pseudotarget disease, the results of arm onewill show the real acupuncture effect, while the results of arm two will demonstrate a combination of the real and placebo effect. The actual effect size of acupuncture for both diseases and the respective placebo effect size can be determined through comparison.

### Key techniques

#### Selection of target disease and pseudotarget disease

The selection of an appropriate target disease and determination of a pseudotarget disease is key to the successful implementation of the design. A target disease should be selected according to the aim of the study. Interventions for the target disease, including acupoints chosen and methods of manipulation, should be planned accordingly. When choosing a pseudotarget disease, the intervention aiming for the target disease shouldproduce no effects on the pseudotarget disease, and vice versa. The clinicians should incorporate traditional Chinese medicine (TCM) theories with clinical experience before reaching a decision.

According to the TCM theories, the various signs and symptoms that one patient presents can be attributed to the same TCM pattern or a correlated set of patterns. It is possible that there may be certain associations or links between the target disease and the pseudotarget disease. However, provided different psychological responses are induced in the two groups, the utility of the TIGER design will not be affected.

Besides that, the feasibility of the study shall be of great concern. To ensure successful progress of the study, researchers should ensure that there are sufficient patients simultaneously suffering from both diseases of interest to yield results of statistical significance.

#### Rigorous management of research staff

To ensure the quality of clinical trials, all participating researchers should be responsible for a specific part of the study. Trial designers, clinical investigators, clinicians, patients, assessors, and statisticians should be independent of each other. The trial designer is responsible for devising a rigorous, scientific, and feasible protocol of the study. Clinical investigators are in charge of patient recruitment, randomization, treatment allocation, and communications with the patients regarding what medication to take and how. Clinicians should administer treatment to patients according to the study protocol, and should not be involved in outcome evaluation. Clinical assessors evaluate patients’ outcomes, and the statisticians are responsible for data analysis.

#### Selection of sensitive outcome measures

The selection of outcomes for clinical evaluation of efficacy or safety should be based on the characteristics of the target disease and the pseudotarget disease. They should possess a certain degree of sensitivity and specificity.

### An example

We plan to run a clinical study of acupuncture therapy targeting migraine headaches, with constipation as the pseudotarget disease. Specifically, participants suffering from migraine and constipation at the same time will be included and randomly assigned to arm one or arm two. Patients in arm one will be informed that they will receive acupuncture therapy for migraine, while patients in arm two will be told they will receive acupuncture for constipation. Acupuncturists who have been practicing for over 10 years will administer the following needling therapy to all participants. Acupoints for migraine headaches chosen by literature search and expert experienceare: Touwei (ST8), Shuaigu (GB8), Yifeng (SJ17), Fengchi (GB20), and Temple (HN5). Needles will be inserted into the above points and will be manipulated using the neutral reinforcing-reducing technique of evenly distributing twirling-rotating and lifting-thrusting of the needle, and will be retained for 30 minutes after the ‘Deqi’ response is elicited. The treatment will be provided once a day for 3 consecutive days.

Upon completion of the treatment, an independent assessor will evaluate the outcome measures and determine whether the same therapy produces different effects in the two arms of participants.

The rationale underlying the TIGER designis that the results of arm one will show both the real acupuncture effects and the placebo effects, while the results of arm two will show only the real acupuncture effects (Table 1).

**Table 1:** Evaluation of the intervention effects in an acupuncture trial using the TIGER design

**Target disease**	**Arm one**	**Arm two**
Migraine	Real acupuncture effects plus placebo effects^a^	Real acupuncture effects^b^
Constipation (pseudo)	Real acupuncture effect^c^	Real acupuncture effects plus placebo effects^d^

^abcd^Represent the effective rate of the four analytical units. TIGER, target disease-guided placebo-controlled.

## Results and discussion

Using the proposed TIGER design, it is possible to observe the specific and placebo effects of acupuncture for migraine and constipation, respectively. However, findings from the assessment of outcomes of arm two are the focus of this study.

While the effects of acupuncture therapy are more or less influenced by patients’ perceptions of needle insertions, we believe that guiding patients’ psychological responses (which has been depicted as a major source of the placebo effects) to a pseudotarget disease using this method may help to reveal the real effects of needling for the target disease.

### Advantages

#### Identical interventions

The same treatment will be given to the two groups of patients (inserting needles into the same acupoints with identical manipulations untilthe same type of ‘Deqi’ response is provoked), thus dispelling confounding doubts from the participantsabout the authenticity of the treatment (that is, whether they received the real treatment) and from varied perceptions of the needling stimulations.

#### Blinding

Acupuncturists will be perfectly blinded as the treatment given to the two arms will be identical. Moreover, patients in arm two will be blinded of the real target disease.

#### Ethical issues

In clinical medicine, the use of placebo treatment for patients has been subject to controversy for years. With the help of the TIGER design, both groups of patients can receive actual acupuncture treatment and their rights will be safeguarded.

### Limitations

There are three main limitations to the TIGER design: 1)the new control design needs to be tested for feasibility in clinical trials for a variety of conditions and diseases. We believe the design can be improved, and the best way is to adapt it in practice; 2) the use of this control design requires the patients to have both the target disease and the pseudotarget disease at the same time. Apart from that, interventions for the target disease should have no therapeutic effects on the pseudotarget disease. Therefore, recruiting qualified patients will be challenging; and 3) the external validity of trial results is invariably affected as participants included are highly selective. In other words, research findings from patients suffering from a combination of conditions may not be applicable to the general patient population. Thereforeresults must be interpreted cautiously.

## Conclusions

Acupuncture therapy has been practiced in China for thousands of years, it has stood the test of time, and it is widely acclaimed for its efficacy and safety. Until recently, the evaluation of acupuncture in a scientific trial design has been a highpriority in the cause of TCM modernization. Skeptical about the therapeutic effects of acupuncture, some have asserted that acupuncture effects are no more than placebo effects. To examine the net specific effects of needling and the placebo effects separately has challenged scholars interested in acupuncture research for decades. In response to this challenge, we developed the TIGER design.

It is worth noting that every type of acupuncture placebo has its own limitations. Whether it is appropriate for use in a specific context largely depends on the aim of the study. The proposed TIGER design is applicable to studies focusing on the specific effects of acupuncture, devoid of non-specific placebo effects.

## Abbreviations

EBM: Evidence-based medicine; RCT: Randomized controlled trial; TCM: Traditional Chinese medicine; TENS: Transcutaneous electrical nerve stimulation; TIGER: Target disease-guided placebo-controlled.

## Competing interests

The authors declare that they have no competing interests.

## Authors’ contribution

WZ conceived the design and drafted the manuscript. HW participated in the study design and helped to draft the manuscript. WM and LZ helped to draft the manuscript. ZB and HS developed the design and supervised the research project. All authors read and approved the final manuscript.

## Authors’ information

WZ is a doctoral candidate of Tianjin University of Traditional Chinese Medicineand has been engaged in the research of clinical evaluation of traditional Chinese medicine for 6 years. HW is a lecturer of medical ethics and EBM at Tianjin University of Traditional Chinese Medicine. WM and LZ are doctoral candidates of Tianjin University of Traditional Chinese Medicine and engage in EBM research. ZB is a professor at Hong Kong Baptist Universityand has spent part of his academic life in clinical trial methodological research for TCM. HS is a professor at Tianjin University of Traditional Chinese Medicine and also director of the Tianjin Center for Evidence-Based Medicine.
